# Cognitive Loading During and After Continuous Task Execution Alters the Effects of Self-Controlled Knowledge of Results

**DOI:** 10.3389/fpsyg.2020.01046

**Published:** 2020-06-03

**Authors:** Kaylee F. Woodard, Jeffrey T. Fairbrother

**Affiliations:** Motor Behavior Laboratory, Department of Kinesiology, Recreation, and Sport Studies, The University of Tennessee, Knoxville, Knoxville, TN, United States

**Keywords:** self-control, feedback, motor performance, motor learning, closed-loop control, information processing, online processing

## Abstract

Previous research has repeatedly demonstrated that providing learners with self-control (SC) over their feedback schedules enhances motor skill learning. Increased information processing under SC conditions has been shown to contribute to these benefits. However, the timing of critical information processing for SC participants during the acquisition of continuous tasks is unknown. The present study was designed to enhance clarity related to this issue. Participants learned a continuous tracing task under SC or yoked (YK) conditions. Groups of participants also completed a secondary cognitive load task either during or after the execution of each primary task trial. Results showed enhanced learning for SC compared to YK participants who did not complete the cognitive load task. However, this benefit was eliminated for SC participants who completed the cognitive load task either during or after the primary task. These findings suggest that effective information processing both during and after continuous task execution is critical for reaping the benefits of self-controlled practice. Further interpretations and implications of these findings as well as suggestions for future research are discussed.

## Introduction

The benefits of providing learners with control over their practice experience have been widely supported in motor learning research. Participants who are given self-control (SC) over some aspect of practice typically experience significant learning advantages compared to those who are not given control (for a review see Wulf, 2007). In research examining this effect, participants have been given control over a variety of practice components including the amount of practice (Post et al., 2011, 2014), the level of task difficulty (Andrieux et al., 2012), observation (Wulf et al., 2005), the use of assistance devices (Wulf and Toole, 1999), and even seemingly trivial details such as the color of practice equipment (Lewthwaite et al., 2015, Experiment 1; Wulf et al., 2018). However, the most common manipulation in this line of research involves giving learners control over feedback schedules. Learners who choose when they receive knowledge of performance (KP) or knowledge of results (KR) consistently show superior learning than those whose feedback schedules are externally controlled (e.g., Chiviacowsky and Wulf, 2002; Aiken et al., 2012; Post et al., 2016). The self-controlled learning benefit has been observed in many different types of discrete and serial tasks including object projection tasks (e.g., Janelle et al., 1995; Fairbrother et al., 2012; Post et al., 2014), anticipation timing tasks (e.g., Ali et al., 2012), and sequencing tasks (e.g., Patterson et al., 2011; Wu and Magill, 2011; Kaefer et al., 2014). More recently, the self-controlled learning effect has been tested in continuous tasks requiring the closed-loop control processes (e.g., Marques and Correa, 2016; Couvillion et al., 2019). Results suggest that SC over outcome-based feedback still significantly affects the learning of such tasks, even though they require online information processing for execution.

Although the benefits of self-controlled practice are robust and span a variety of task types, questions still remain about the mechanisms that underlie this effect. The motivation perspective and the information processing perspective have been forwarded as the two primary explanations for the SC effect. While it is possible, and even quite likely, that enhanced motivation and information processing work in tandem to produce learning benefits, most research has examined these mechanisms in isolation. The motivation perspective suggests that providing learners with control over their practice experience supports the fulfillment of basic psychological needs, thus enhancing intrinsic motivation, and promoting learning (Ryan and Deci, 2000; Chiviacowsky et al., 2012; Sanli et al., 2013). Studies showing that participants tend to request feedback after relatively good trials suggest that learners may use their autonomy to enhance or protect feelings of competence (e.g., Chiviacowsky et al., 2008; Fairbrother et al., 2012). Results from post-training questionnaires have also demonstrated enhanced self-efficacy ratings (e.g., Chiviacowsky, 2014) as well as direct increases in intrinsic motivation when SC is given (e.g., Grand et al., 2015). Other research, however, has noted that the good-trial preference may be stronger in later stages of practice (Carter and Patterson, 2012) and may have been driven by different task demands (e.g., simplicity of the tasks) and feedback used in previous research (Aiken et al., 2012). Further, studies have shown that SC participants make feedback choices based on a variety of strategies, rather than simply based on the quality of the trial (e.g., Laughlin et al., 2015; Carter et al., 2016). The motivation perspective has also been supported in studies demonstrating learning benefits for participants who are given choices regarding task-irrelevant features such as laboratory decorations or the color of practice gear (Lewthwaite et al., 2015). It must be noted, however, that other studies have shown that task-relevant choices offer greater learning benefits compared to task-irrelevant choices (Carter and Ste-Marie, 2017b). In fact, these authors found no differences on retention and transfer tests between groups who were given a task-irrelevant choice versus no choice at all, indicating that enhanced motivation does not fully account for self-controlled learning benefits.

The information processing perspective suggests that learning benefits under self-controlled practice conditions are likely due to improved processing of task relevant information (Janelle et al., 1995, 1997). This idea has been indirectly supported through research showing that self-controlled participants engaged in longer preparation times before practice trials and had better recall of practice details compared to yoked (YK) participants (Post et al., 2011). A number of studies have also provided more direct support for the information processing perspective. For example, Chiviacowsky and Wulf (2005) tested learning in two separate groups who were given control over KR at distinct time periods. One group (self-after) followed the traditional pattern of choosing whether to receive feedback for each trial after the completion of that trial. The second group (self-before) was required to choose whether or not they wanted feedback *prior* to beginning each trial, preventing these participants from using performance-related information to make feedback decisions. Results showed that while both groups improved their performance throughout acquisition, the self-after group was superior to the self-before group in the transfer test. The transfer test in this study required participants to perform the original task with an alteration in overall movement time. Thus, superior performance on this task indicate that the self-after group developed a stronger representation of the criterion task and capability for adapting to new parameters compared to the self-before group. A similar study by Carter et al. (2014) replicated and extended this finding. In addition to self-before and self-after groups, Carter et al. (2014) included a third group of participants (self-both) who made feedback decisions before each trial but were allowed to change their decision after completing the trial. In this study, the self-both and self-after groups each displayed enhanced learning compared to their YK counterparts and compared to the self-before group. Together, the results of these studies provide support for the importance of using performance-related information to make effective feedback decisions and reap the learning benefits of self-controlled practice.

Some more recent studies in this line of research have focused on disrupting participants’ capacity for information processing during practice. Specifically, Carter and Ste-Marie (2017a) sought to manipulate the allocation of attentional resources in the period of time between completing each trial and requesting feedback (i.e., the KR-delay interval). Two SC groups and corresponding YK groups learned an elbow extension-flexion task either with or without the presence of an interpolated event during the KR-delay interval. The interpolated event was a number identification task (cf. Marteniuk, 1986) designed to engage attentional resources and hinder learners from processing performance-related information from their previous trial. Results showed that the self-controlled learning benefit was eliminated for the group that completed the interpolated event, thus demonstrating the importance of information processing during the KR-delay interval for benefitting from the provision of SC over feedback.

The aforementioned studies have all examined the information processing perspective and self-controlled learning using open-loop, discrete tasks. For this type of task, processing of performance related information is thought to occur almost exclusively after trial execution. However, continuous tasks such as driving or writing often require the performer to use online closed-loop control processes to use information during task execution. Given the practical significance of such tasks and the distinct nature of information processing required for successful performance, it is important to examine the relationship between SC effects and continuous, online information processing. Couvillion et al. (2019) examined this relationship in a tracing task using a protocol based on Carter and Ste-Marie’s (2017a) experiment. Two pairs of SC and YK groups learned to trace a star-shaped pattern either with or without an additional cognitive load (i.e., a number identification task) imposed during acquisition. To reduce the attentional capacity that was available for online processing, participants were required to complete the number identification task *during* execution of the primary tracing task. Results showed that the self-controlled learning benefit was eliminated in groups who performed the cognitive load task, thus demonstrating the importance of online information processing in demonstrating a SC feedback effect. However, it is still unknown whether information processing during task execution is the only, or the most important, factor related to self-controlled feedback effects in continuous tasks. Couvillion et al. pointed out that participants who completed the cognitive load task likely had impaired information processing capability both during and after execution of the primary tracing task. That is, completing the number identification task presumably degraded participants’ capacity for online information processing, and this online degradation likely reduced the availability of performance-related information for subsequent processing during the KR-delay interval. Therefore, it is still unknown whether the SC effect was eliminated in this study due to impaired information processing *during* task execution or due to a lack of availability of performance-related information for processing *after* task execution (i.e., during the KR-delay interval). Further work is necessary to determine the most critical time period of information processing for producing self-controlled feedback effects.

The purpose of the present experiment was twofold. First, we sought to replicate the results of our previous study (Couvillion et al., 2019) and second, to determine the role of processing information during the KR-delay interval for demonstrating a self-controlled feedback effect for a continuous task. If information processing during the KR-delay interval is critical for SC effects, then introducing a load during this period should eliminate the effect even when a load was not experienced during execution. If effective information processing is critical both during execution and the KR-delay, then SC effects should be eliminated regardless of when the load was introduced.

## Materials and Methods

### Participants

Participants were 72 students (16 males, 56 females, 20.38 ± 1.67 year) at a university in the southeast United States. All participants reported having no prior experience with the experimental task and provided voluntary informed consent prior to engaging in any research activities.

### Task

The experimental task involved tracing a star pattern with a stylus which was attached to two multi-joint lever-arms (Two-Arm Coordination Test; Lafayette Instrument Model 32532, Lafayette, IN, United States). The Two-Arm Coordination Test instrument was modified by the addition of two telegraph keys to start and stop the timer, thereby requiring movement of the hands from the keys to the levers at the start and back to the keys at the end. Participants learned to control the lever-arms so that the stylus moved in a clockwise direction along the anodized star pattern. The goal of the task was to trace the entire star pattern as quickly as possible while committing the fewest errors possible.

**FIGURE 1:**
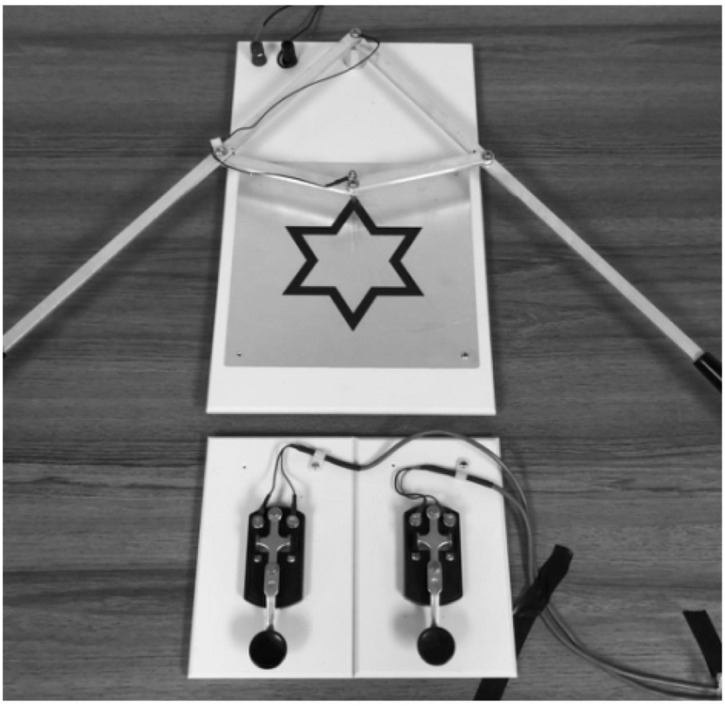
Diagram of apparatus.

A depiction of the apparatus is shown in Figure 1. The Two-Arm Coordination Test was connected to two telegraph keys, designated as *Start* and *Stop* keys, and a Multi-Function Timer/Counter (Lafayette Instrument Model 54035A, Lafayette, IN, United States) which was used to record movement time and errors. Prior to each trial, the participant used their preferred hand to depress the telegraph key on the left-hand side (i.e., the *Start* key). The trial began when the participant released the key (to move the hands to the handles of the lever arms) and ended when they depressed the *Stop* key located on the right-hand side (after finishing the tracing and removing the hands from the handles). Movement time was the elapsed time between release of the *Start* key and depression of the *Stop* key. An error was counted each time the metal stylus moved off of the anodized pattern and onto the surrounding metal plate. For the transfer test, the tracing task was modified such that the star path was approximately 50% narrower than the original pattern (i.e., reduced from 0.95 to 0.48 cm). The total length (19.5 cm) remained unchanged from the retention to the transfer test.

During acquisition, the load groups (SC_LD, YK_LD, SC_LA, and YK_LA) also completed a secondary cognitive load task. The secondary task required participants to identify randomly generated three-digit numbers through continuous verbal exchange with the researcher (cf. Marteniuk, 1986; Carter and Ste-Marie, 2017a; Couvillion et al., 2019). To complete this task, the participant was asked to guess an initial three-digit number. The researcher then responded with a combination of the words “high,” “low,” and “spot” to indicate whether each digit of the guessed number was higher, lower, or the same as the corresponding digit of the correct number. Based on this response, the participant made another guess, and this exchange continued for the allotted time period. In cases when the participant determined the correct number before the allotted time was up, the researcher immediately prompted them to begin guessing a new number for the remainder of the time period.

### Procedure

Participants completed two research sessions on two consecutive days. Upon arrival to the laboratory, participants provided informed consent, and were quasi-randomly assigned to one of six experimental groups: SC (3 males, 9 females, 20.82 ± 2.32 year), YK (4 males, 8 females, 20.42 ± 1.62 year), Self-Control + Load During (SC_LD) (3 males, 9 females, 20.67 ± 0.65 year), Yoked + Load During (YK_LD) (1 males, 11 females, 19.75 ± 0.97 year), Self-Control + Load After (SC_LA) (3 males, 9 females, 20.92 ± 2.5 year), and Yoked + Load After (YK_LA) (2 males, 10 females, 19.75 ± 1.06 year). Group sample sizes (*n* = 12) were determined based on approximations from previous literature on cognitive loading and self-controlled feedback effects (i.e., Carter and Ste-Marie, 2017a; Couvillion et al., 2019). The University of Tennessee Institutional Review Board approved all study procedures and the informed consent document.

All participants were shown the apparatus and given instructions about how to complete the task. They were told to learn to trace the star-shaped pattern by moving the two handles that were attached to the stylus. No further instructions were given regarding the specific operation of the handles. Participants were told that the goal of the task was to trace the star as quickly and accurately as possible. Speed and accuracy were emphasized equally. They were instructed to begin and end each trial by pressing the telegraph keys, which were located in front of the star-tracing apparatus on the left and right sides, respectively. Participants in the SC, SC_LD, and SC_LA groups were told that they could ask for feedback after any practice trial, and that the feedback would consist of their movement time and error score for that trial. Participants in the YK, YK_LD, and YK_LA groups were told that they would periodically receive feedback on their movement time and error scores after certain trials. All feedback was presented verbally immediately upon completion of the trial (or immediately upon request for self-controlled groups). Each feedback presentation included movement time followed by the error score. Directly following feedback presentation, the timer was reset, and the participant was verbally prompted to begin the next trial. This resulted in approximately 3 s to process feedback before the next trial. A standard yoking procedure was used, such that the feedback schedule for participants in the YK groups was determined by their SC counterparts. Specifically, the FB schedule for the YK group was determined by the SC group, for the YK_LD group by the SC_LD group, and for the YK_LA group by the SC_LA group. Participants were paired randomly based on the order in which they completed the study. Participants in the loaded groups were also given instructions about the secondary number-guessing task and told that they would be completing this task during (SC_LD and YK_LD groups) or after (SC_LA and YK_LA groups) each trial of the primary task. Participants in the SC_LD and YK_LD groups performed the secondary task for the entire duration of each primary task trial. Participants in the SC_LA and YK_LA groups performed the secondary task for a period of 15-s directly following each primary task trial. The intertrial interval was approximately 10 s for SC, YK, SC_LD and YK_LD, respectively groups, and approximately 25 s for the SC_LA and YK_LA, respectively groups due to the additional time required to complete the cognitive load task following each trial. The inter-block interval was approximately 1 min for all groups.

During Session 1, participants completed the acquisition phase which consisted of four blocks of eight trials. On the following day, participants returned to the lab to complete retention and transfer testing. The retention test consisted of one block of eight trials on the original task. The transfer test consisted of one block of eight trials on the narrower version of the tracing task. All procedures during the retention and transfer tests were identical to acquisition, with the exception that no feedback was given and no secondary task was completed during testing.

### Data Treatment and Analysis

The primary dependent variables of interest were movement time (MT) and Errors. MT was defined as the time (s) between the release of the *Start* button and depression of the *Stop* button for each trial. Errors were defined as the number of times the stylus deviated from the anodized star path and onto the metal plate during each trial. Participants’ MT and Errors were recorded for each trial and averaged across eight trials for each acquisition block. Average MT and Errors were then analyzed using separate 2 (FB schedule) × 3 (load) × 4 (block) analyses of variance (ANOVA) with repeated measures on the last factor. For the retention and transfer tests, MT, and Errors were averaged across blocks of eight trials and analyzed using separate 2 (feedback schedule) × 3 (load) ANOVAs. The number of feedback requests for the SC, SC_LD, and SC_LA groups were tabulated across acquisition blocks and analyzed using a Chi-Square test of independence. Similarly, the number of correct secondary task responses for the SC_LD, YK_LD, SC_LA, and YK_LA groups were tabulated for each acquisition block and reported descriptively. Cook’s distance (Cook, 1977; Cook and Weisberg, 1999) was used to check for any outliers in the dataset. Tests of normality revealed that MT data were not normally distributed in some cases. However, ANOVA is considered to be robust to non-normality (Blanca Mena et al., 2017). The Greenhouse-Geisser *df* adjustment was used to handle violations of sphericity in all repeated measures analyses. Bonferroni procedures were used to determine all *post hoc* comparisons. Alpha was set to 0.05 for all statistical comparisons.

## Results

### Acquisition

#### Feedback Request Frequency

The overall feedback request frequency was 31% (118 requests) for both the SC and SC_LA groups and 19% (72 requests) for the SC_LD group. A Chi-Square test of independence was performed to analyze the relationship between group and total number of feedback requests across practice. The Chi-Square was significant (*p* < 0.001), showing that the SC_LD group was less likely to request feedback compared to the SC and the SC_LA groups. For the SC group, feedback request frequency was lowest in the first two blocks (24 and 23%, respectively) and highest in the last two blocks (35% and 41%, respectively). For the SC_LA group, request frequency was lowest in the first block (23%), increased slightly in blocks 2 and 3 (28 and 29%, respectively), and was highest in the final block (41%). For the SC_LD group, a slightly different pattern emerged, such that feedback request frequency slightly decreased from block 1 to block 4 (21–17%, respectively).

#### Secondary Task Performance

Participants achieved between 0 and 1 correct secondary task responses on the majority of trials. Specifically, participants in the SC_LA and YK_LA groups made between 0 and 1 correct responses on 94 and 98% of trials, respectively. Participants in the SC_LD and YK_LD groups made between 0 and 1 correct responses on 73 and 87% of trials, respectively.

#### Movement Time

**FIGURE 2:**
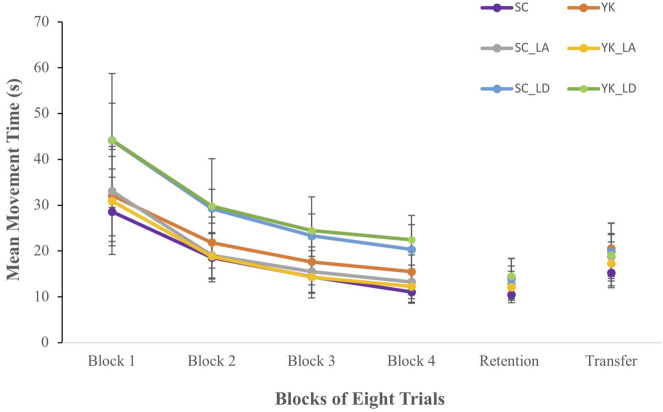
Mean movement time scores for each group during each block of acquisition, retention, and transfer.

Mean MT scores for each group and each acquisition block are shown in the left panel of Figure 2. Overall, MT decreased across blocks and was longer for SC_LD and YK_LD groups compared to all other groups. These observations were supported by a significant block x load interaction, *F*(6,198) = 3.55, *p* = 0.025, $\eta_{p}^{2}$ = 0.097. *Post hoc* procedures revealed that participants in the Load-During condition had significantly longer MT scores than participants in all other conditions in all four blocks. Additionally, MT was shorter for all participants in each block compared to the previous block (*p* < 0.001). The main effects for block, *F*(3,198) = 412.95, *p* < 0.001, $\eta_{p}^{2}$ = 0.097, and load, *F*(2,66) = 21.85 *p* < 0.001, $\eta_{p}^{2}$ = 0.398 were also significant.

#### Errors

**FIGURE 3:**
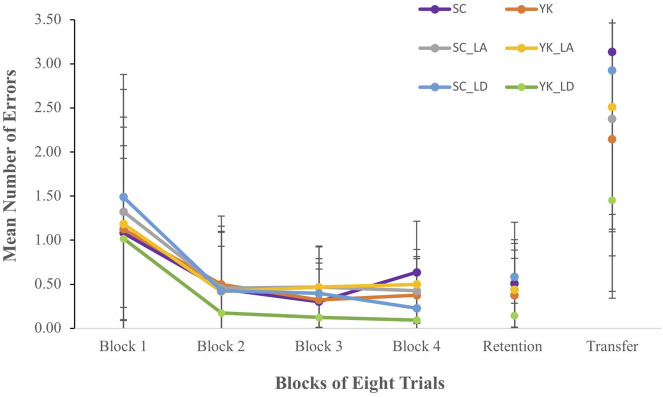
Mean error scores for each group during each block of acquisition, retention, and transfer.

Mean Errors for each group and each acquisition block are shown in the left panel of Figure 3. For all groups, Errors were highest in block 1 compared to blocks 2–4. This observation was supported by a significant main effect for block, *F*(3,198) = 38.59, *p* < 0.001, $\eta_{p}^{2}$ = 0.369. *Post hoc* procedures revealed that Errors were significantly higher in block 1 compared to all other blocks (*p* < 0.001). No other main effects or interactions were significant.

### Retention and Transfer

#### Movement Time

Mean MT during retention and transfer tests for each group are shown in the right panel of Figure 2. During both retention and transfer tests, mean MT was lowest for the SC group. This observation was supported by a significant FB schedule x load interaction in retention, *F*(2,66) = 3.807, *p* = 0.03, $\eta_{p}^{2}$ = 0.103 and in transfer, *F*(2,66) = 3.677, *p* = 0.031, $\eta_{p}^{2}$ = 0.103. *Post hoc* comparisons revealed that within the No Load condition, participants in the SC group performed significantly faster than those in the YK group during the retention test (*p* = 0.002) and the transfer test (*p* = 0.008). MT did not differ between the SC and YK groups within either the Load-During or Load-After conditions during retention (*p* = 0.64 and 0.525, respectively) or transfer (*p* = 0.736 and 0.414, respectively). No other comparisons or interactions were significant.

#### Errors

Mean Errors during retention and transfer tests for each group are shown in the right panel of Figure 3. In the retention test, Errors were similar between all groups, whereas in the transfer test, Errors were slightly higher for the SC_LD group compared to all other groups. Overall, Errors were higher in the transfer test compared to the retention test. These observations were partially supported by a main effect for FB schedule in transfer, *F*(1,66) = 4.039, *p* = 0.049, $\eta_{p}^{2}$ = 0.058. Group mean comparisons revealed that participants in the SC condition committed significantly more Errors (*M* = 1.67, *SD* = 2.22) than participants in the YK condition (*M* = 1.18, *SD* = 1.85). No other comparisons or interactions were significant.

## Discussion

Research has consistently shown that providing learners with SC over their practice experience enhances learning, but questions remain regarding the roles of motivation and information processing in producing this effect (for a review see Sanli et al., 2013). Additionally, the nature and timing of critical information processing for SC participants is still unclear. The present study was designed to investigate the impact of information processing during two time periods, namely during and after the execution of a continuous task. Results replicated and extended previous findings (Couvillion et al., 2019), demonstrating that information processing both during and after continuous task execution is influential in producing self-controlled feedback effects.

Distinct patterns of FB request frequencies emerged among the three SC groups. Participants in the Load During condition were significantly less likely to request feedback across practice compared to those in the No Load and Load-After conditions. Additionally, while participants in the SC_LD group followed a faded FB schedule, aligning with the patterns reported in prior research (e.g., Ali et al., 2012), FB request frequencies for participants in the No Load and Load-After conditions tended to increase across blocks. Perhaps the task and feedback used in the current study were simple enough to expect that participants may have preferred receiving FB after trials that they perceived to be good (Chiviacowsky and Wulf, 2002; Aiken et al., 2012; Fairbrother et al., 2012) and that these trials increased in frequency as performance improved. Granted, this explanation only accounts for the patterns of FB requests observed in No Load and Load-After groups. It is unclear why participants in the Load-During condition displayed distinct FB request patterns.

All groups showed similar patterns of improvement across acquisition in both MT and Error measures. No between-group differences were observed in acquisition, except that MT was longer for those in the Load-During condition compared to all other conditions. This slowing was expected and demonstrates that the cognitive load task was successful in inducing an increased information processing load during task execution. That is, participants in the Load-During condition presumably had to slow their movement in order to process the additional information related to the secondary task while also completing the online processing required for success in the primary task. However, it does not appear that this increased burden impeded learning altogether, as all groups showed significant improvements during acquisition and maintained these improvements in subsequent retention testing. It is interesting to note that the frequency of FB requests varied across SC groups, which limits comparisons across these groups in retention and transfer. However, the central question in the present study is related to the comparisons between each SC group and its YK counterpart. Due to the yoking procedure that was used, FB frequencies were identical for each pair of SC and YK groups, and thus, FB frequency would have no bearing on any learning differences that emerged within these pairings.

Learning differences between the traditional SC and YK groups emerged in MT scores. SC participants performed significantly faster than YK participants in the No Load condition, but there were no differences between SC and YK participants in the Load-After or Load-During conditions. Thus, the SC effect was eliminated when attention was disrupted either during or after execution of the primary task, suggesting that the information processed during both of these time periods was important for demonstrating the self-controlled feedback effect. This pattern was consistent with previous results (Couvillion et al., 2019).

Analysis of the number of Errors, however, revealed unexpected findings. Participants who were given SC committed significantly more Errors compared to their YK counterparts in transfer, regardless of whether or not they performed the additional cognitive load task. Thus, the provision of SC over FB produced a detriment with respect to accuracy under the heightened demand of the transfer test. This result was in contrast to our expectations and did not align with most previous research on SC over feedback (e.g., Janelle et al., 1997; Chiviacowsky et al., 2008). It is also important to note that even though SC participants committed a statistically greater number of Errors than the YK participants, this effect was isolated in the transfer test, and the numerical difference between groups was less than one Error. Whether this accuracy detriment negates the overall benefit of SC over FB is dependent upon context. For example, in the current study’s tracing task, the practical importance of this accuracy difference seems minimal, whereas in other situations such as a surgical procedure, even one error may be catastrophic. Moreover, what the current finding illustrates is that SC produces effects that may or may not be considered beneficial depending upon task demands. Future research should move beyond a search for evidence to support a preconceived notion that self-controlled practice conditions are always beneficial to learning.

Accounting for both MT and Errors, the current results indicate behavioral *differences* between participants in SC and YK conditions and distinct responses to the SC manipulation in the Load vs. No Load conditions. The provision of SC in the No Load condition manifested a benefit in one dimension (i.e., MT) and a detriment in another dimension (i.e., Errors) during transfer, whereas SC in both loaded conditions produced an accuracy detriment in transfer *without* the accompanying MT benefit. Thus, it is still evident that the processing of information both during and after continuous task execution is relevant for making FB decisions, even if SC over feedback does not produce clear learning benefits. This finding supported previous research (Couvillion et al., 2019) showing the importance of information processing during the execution of a continuous task. Additionally, the current results added to the existing body of literature by demonstrating that a cognitively demanding task performed during the KR-delay interval was also influential in eliminating SC FB effects.

These findings raise questions regarding the specific nature of the information being processed during each time interval. One possibility is that that the load-after and load-during manipulations affected two distinct types of information, both of which were critical for producing the traditional SC effect. Another, perhaps more plausible, explanation is that the information normally processed during and after task execution is similar in content, but that the timing of the cognitive load task in the current study differentially impacted participants’ ability to use this information. According to this logic, the load-during conditions would have presumably hindered online processing of subjective task-related information, rendering the information *unavailable* for post-execution processing, whereas the load-after condition would have simply blocked *access* to this information during the time when it was needed for making FB decisions (i.e., the KR-delay interval). Thus, the loaded conditions would have disrupted either the generation (load-during condition) or the effective use (load-after condition) of the same informational content. This explanation is consistent with concepts from Schema Theory (Schmidt, 1975) in that subjective reinforcement occurring *after* discrete task execution is presumed to draw upon the same information that is generated *during* execution. Of course, this explanation is still speculative with regard to the current study and in the context of a continuous task, thus highlighting the need for further research.

As discussed previously, SC participants in the current study displayed distinct behavioral patterns with respect to MT and accuracy. Spontaneous verbal responses during acquisition and the nature of the experimental task seem to provide logical explanations for these behavioral differences. Even though participants knew that any request for FB would yield information about both MT and Errors, SC participants often asked specifically for their MT scores but rarely asked specifically for their Error scores. Correspondingly, given that participants could presumably see when the stylus left the designated track, the most salient information presented in the augmented feedback was likely MT rather than accuracy scores. Along this line of reasoning, it is plausible that SC participants made FB decisions based on the need to ascertain unknown information about their speed (i.e., MT), and thus were encouraged to use this information for improving performance in this measure. This explanation is compatible with other research suggesting that requested FB is most beneficial when it provides information that reduces uncertainty about the quality of performance (e.g., Carter et al., 2014). It also aligns well with the information processing perspective. Specifically, if participants made FB decisions for the primary purpose of gaining information about MT, and if the cognitive load task hindered SC participants from making meaningful FB decisions, then only SC participants in the No Load condition should reap learning benefits expressed in the MT measure. This pattern was indeed demonstrated in the retention and transfer results. Interestingly, a similar emphasis of movement speed emerged during the acquisition phase in prior research on this task (Couvillion et al., 2019). It is unclear why this tradeoff emerged during distinct phases in the two studies, but the general pattern of emphasizing speed under SC conditions merits further investigation.

The results of the current study demonstrated initial evidence that SC over feedback can cause participants to prioritize speed over accuracy, particularly when information processing is compromised. As such, practitioners should evaluate the relative importance of accuracy compared to speed and similar performance measures when making decisions regarding feedback schedules. Specifically, for motor skills in which mistakes can lead to disqualification or even injury (e.g., faulting in long jumping, lane deviation in driving, and falls in gymnastics skills), practitioners may need to reconsider providing SC over feedback during practice. Future research is warranted to provide further clarity on the effects of SC for tasks involving competing performance measures.

### Limitations

Although the current study provided strong contributions to the current understanding of self-controlled FB effects, there are a few limitations to consider. First, the study did not provide insight into the specific *content* of information which was affected in the load-after and load-during conditions. A second limitation of the study is the small sample size. Although the sample size was presumably large enough to accurately detect SC effects, a larger sample size may have allowed for greater differentiation of participants’ responses to the load-after and load-during conditions. Future research will be needed to address both of these limitations and provide further insight into the specific influence of information processing on self-controlled FB effects.

## Data Availability Statement

The raw data supporting the conclusions of this article will be made available by the authors, without undue reservation, to any qualified researcher.

## Ethics Statement

This study was carried out in accordance with the recommendations of the University of Tennessee Institutional Review Board with written informed consent from all subjects. All subjects gave written consent in accordance with the Declaration of Helsinki. The protocol was approved by the University of Tennessee Institutional Review Board.

## Author Contributions

JF contributed to design and conception of the study. JF and KW analyzed and interpreted the data, contributed to manuscript revision, read, and approved the submitted version. KW wrote the first draft of the manuscript.

## Conflict of Interest

The authors declare that the research was conducted in the absence of any commercial or financial relationships that could be construed as a potential conflict of interest.

## References

[B1] Aiken C. A. Fairbrother J. T. Post P. G. (2012). The effects of self-controlled video feedback on the learning of the basketball set shot. *Front. Psychol.* 3:338. 10.3389/fpsyg.2012.00338 22973257 PMC3438820

[B2] Ali A. Fawver B. Kim J. Fairbrother J. Janelle C. M. (2012). Too much of a good thing: random practice scheduling and self-control of feedback lead to unique but not additive learning benefits. *Front. Psychol.* 3:503. 10.3389/fpsyg.2012.00503 23233843 PMC3517989

[B3] Andrieux M. Danna J. Thon B. (2012). Self-control of task difficulty during training enhances motor learning of a complex coincidence-anticipation task. *Res. Q. Exerc. Sport* 83 27–35. 22428409 10.1080/02701367.2012.10599822

[B4] Blanca Mena B. Alarcón R. Arnau Gras J. Bono Cabré R. Bendayan R. (2017). Non-normal data: is ANOVA still a valid option? *Psicothema* 29 552–557. 10.7334/psicothema2016.383 29048317

[B5] Carter M. Rathwell S. Ste-Marie D. (2016). Motor skill retention is modulated by strategy choice during self-controlled knowledge of results schedules. *J. Mot. Learn. Dev.* 4 100–115. 10.1123/jmld.2015-0023

[B6] Carter M. J. Patterson J. T. (2012). Self-controlled knowledge of results: age-related differences in motor learning, strategies, and error detection. *Hum. Mov. Sci*. 31, 1459–1472. 10.1016/j.humov.2012.07.008 23164628

[B7] Carter M. J. Ste-Marie D. M. (2017a). An interpolated activity during the knowledge-of-results delay interval eliminates the learning advantages of self-controlled feedback schedules. *Psychol. Res.* 81 399–406. 10.1007/s00426-016-0757-2 26892773

[B8] Carter M. J. Ste-Marie D. M. (2017b). Not all choices are created equal: task-relevant choices enhance motor learning compared to task-irrelevant choices. *Psychon. Bull. Rev.* 24 1879–1888. 10.3758/s13423-017-1250-7 28224481

[B9] Carter M. J. Carlsen A. N. Ste-Marie D. M. (2014). Self-controlled feedback is effective if it is based on the learner’s performance: a replication and extension of Chiviacowsky and Wulf (2005). *Front. Psychol.* 5:1325 10.3389/fpsyg.2012.001325 PMC4237043 25477846

[B10] Chiviacowsky S. (2014). Self-controlled practice: autonomy protects perceptions of competence and enhances motor learning. *Psychol. Sport Exer.* 15 505–510. 10.1016/j.psychsport.2014.05.003

[B11] Chiviacowsky S. Wulf G. (2002). Self-controlled feedback: does it enhance learning because performers get feedback when they need it? *Res. Q. Exer. Sport* 73 408–415. 10.1080/02701367.2002.10609040 12495242

[B12] Chiviacowsky S. Wulf G. (2005). Self-controlled feedback is effective if it is based on the learner’s performance. *Res. Q. Exerc. Sport* 76 42–48. 15810769 10.1080/02701367.2005.10599260

[B13] Chiviacowsky S. Wulf G. Laroque de Medeiros F. Kaefer A. Tani G. (2008). Learning benefits of self-controlled knowledge of results in 10-year-old children. *Res. Q. Exer. Sport* 79 405–410. 10.1080/02701367.2008.10599505 18816953

[B14] Chiviacowsky S. Wulf G. Lewthwaite R. (2012). Self-controlled learning: the importance of protecting perceptions of competence. *Front. Psychol.* 3:458. 10.3389/fpsyg.2012.00458 23130006 PMC3487418

[B15] Cook R. D. (1977). Detection of influential observation in linear regression. *Technometrics* 19 15–18. 10.1080/00401706.1977.10489493

[B16] Cook R. D. Weisberg S. (1999). *Relating Mean Functions. Applied Regression Including Computing And Graphics.* New York, NY: Wiley, 263–286.

[B17] Couvillion K. Bass A. D. Fairbrother J. T. (2019). Increased cognitive load during acquisition of a continuous task eliminates the learning effects of self-controlled knowledge of results. *J. Sports Sci.* 38 94–99. 31648607 10.1080/02640414.2019.1682901

[B18] Fairbrother J. T. Laughlin D. D. Nguyen T. V. (2012). Self-controlled feedback facilitates motor learning in both high and low activity individuals. *Front. Psychol.* 3:323. 10.3389/fpsyg.2012.00323 22969745 PMC3431613

[B19] Grand K. F. Bruzi A. T. Dyke F. B. Godwin M. M. Leiker A. M. Thompson A. G. (2015). Why self-controlled feedback enhances motor learning: answers from electroencephalography and indices of motivation. *Hum. Mov. Sci.* 43 23–32. 10.1016/j.humov.2015.06.013 26163375

[B20] Janelle C. M. Barba D. A. Frehlich S. G. Tennant L. K. Cauraugh J. H. (1997). Maximizing performance feedback effectivenss through videotape replay and a self-controlled learning environment. *Res. Q. Exer. Sport* 68 269–279. 10.1080/02701367.1997.10608008 9421839

[B21] Janelle C. M. Kim J. Singer R. N. (1995). Subject-controlled performance feedback and learning of a closed motor skill. *Percept. Mot. Skills* 81 627–634. 8570369 10.1177/003151259508100253

[B22] Kaefer A. Chiviacowsky S. Tani G. (2014). Self-controlled practice enhances motor learning in introverts and extroverts. *Res. Q. Exerc. Sport* 85 226–233. 10.1080/02701367.2014.893051 25098018

[B23] Laughlin D. D. Fairbrother J. T. Wrisberg C. A. Alami A. Fisher L. A. Huck S. W. (2015). Self-control behaviors during the learning of a cascade juggling task. *Hum. Mov. Science* 41 9–19. 10.1016/j.humov.2015.02.002 25706605

[B24] Lewthwaite R. Chiviacowsky Z. Drews R. Wulf G. (2015). Choose to move: the motivational impact of autonomy support on motor learning. *Psychon. Bull. Rev.* 22 1383–1388. 25732095 10.3758/s13423-015-0814-7

[B25] Marques P. G. Correa U. C. (2016). The effect of learner’s control of self-observation strategies on learning of front crawl. *Acta Psychol.* 164 151–156. 10.1016/j.actphs.2016.01.006 26821171

[B26] Marteniuk R. G. (1986). Information processes in movement learning: capacity and structural interference effects. *J. Mot. Behav.* 18 55–75. 15136284 10.1080/00222895.1986.10735370

[B27] Patterson J. T. Carter M. Sanli E. (2011). Decreasing the proportion of self-control trials during the acquisition period does not compromise the learning advantages in a self-controlled context. *Res. Q. Exer. Sport* 82 624–633. 10.1080/02701367.2011.10599799 22276404

[B28] Post P. G. Aiken C. A. Laughlin D. D. Fairbrother J. T. (2016). Self-control over combined video feedback and modeling facilitates motor learning. *Hum. Mov. Sci.* 47 49–59. 10.1016/j.humov.2016.01.014 26874750

[B29] Post P. G. Fairbrother J. T. Barros J. A. C. (2011). Self-controlled amount of practice benefits learning of a motor skill. *Res. Q. Exer. Sport* 82 474–481. 10.1080/02701367.2011.10599780 21957706

[B30] Post P. G. Fairbrother J. T. Barros J. A. C. Kulpa J. D. (2014). Self-controlled practice within a fixed time period facilitates the learning of a basketball set shot. *J. Mot. Learn. Dev.* 2 9–15. 10.1123/jmld.2013-0008

[B31] Ryan R. M. Deci E. L. (2000). Self-determination theory and the facilitation of intrinsic motivation, social development, and well-being. *Am. Psychol.* 55 68–78. 10.1037//0008-066X.55.1.68 11392867

[B32] Sanli E. A. Patterson J. T. Bray S. R. Lee T. D. (2013). Understanding self-controlled motor learning protocols through the self-determination theory. *Front. Psychol.* 3:611. 10.3389/fpsyg.2012.00611 23430980 PMC3576889

[B33] Schmidt R. A. (1975). A schema theory of discrete otor skill learning. *Psychol. Rev.* 82 225–260.

[B34] Wu W. F. W. Magill R. A. (2011). Allowing learners to choose. *Res. Q. Exerc. Sport* 82 449–457. 10.1080/02701367.2011.10599777 21957703

[B35] Wulf G. (2007). Self-controlled practice enhances motor learning: implications for physiotherapy. *Physiotherapy* 93 96–101. 10.1016/j.physio.2006.08.005

[B36] Wulf G. Iwatsuki T. Machin B. Kellog J. Copeland C. Lewthwaite R. (2018). Lassoing skill through learner choice. *J. Mot. Behav.* 50 285–292. 28854061 10.1080/00222895.2017.1341378

[B37] Wulf G. Raupach M. Pfeiffer F. (2005). Self-controlled observational practice enhances learning. *Res. Q. Exer. Sport* 76 107–111. 10.1080/02701367.2005.10599266 15810775

[B38] Wulf G. Toole T. (1999). Physical assistance devices in complex motor skill learning: benefits of a self-controlled practice schedule. *Res. Q. Exer. Sport* 70 265–272. 10.1080/02701367.1999.10608045 10522284

